# False belief understanding in deaf children: what are the difficulties?

**DOI:** 10.3389/fpsyg.2024.1238505

**Published:** 2024-01-18

**Authors:** Marek Meristo, Luca Surian, Karin Strid

**Affiliations:** ^1^Department of Psychology, University of Gothenburg, Gothenburg, Sweden; ^2^Department of Psychology and Cognitive Science, University of Trento, Trento, Italy

**Keywords:** social cognition, deafness, cognitive development, eye tracking, theory of mind

## Abstract

Children with cochlear implants (CIs) demonstrate proficiency in verbal-story elicited-response (*VS*-ER) false-belief tasks, such as the Sally & Ann task, at a similar age as typically developing hearing children. However, they face challenges in non-verbal spontaneous-response (NV-SR) false-belief tasks, measured via looking times, which hearing infants typically pass by around 2 years of age, or earlier. The purpose of the present study was to examine whether these difficulties remain in a non-verbal-story elicited-response (NVS-ER) false-belief task, in which children are offered the opportunity to provide an elicited response to a non-verbal-story task. A total of thirty 4- to 8-year-old children with CI-s and hearing children completed three different kinds of false-belief tasks. The results showed that children with CI-s performed above chance level on the verbal task (i.e., *VS*-ER task), but not on the two non-verbal tasks, (i.e., NVS-ER and NV-SR tasks). The control group of typically developing hearing children performed above chance on all three kinds of tasks (one-tailed significance level). Our findings highlight the importance of external narrative support for children with CIs in tasks that involve mental perspective-taking, and specifically predicting actions based on false beliefs.

## Introduction

Understanding that others have intentions, emotions, and beliefs and using that understanding to explain and predict their behavior is crucial for our social interactions. Children are often regarded as having achieved an important milestone in their social development when they can attribute false beliefs (FB) to other individuals, demonstrating their ability to use metarepresentations to understand others’ actions, communications and mental lives. This ability is typically tested by using what we can call classic verbal-story elicited-response (*VS*-ER) false-belief tasks, where children are presented with a verbal story about a character who holds a false belief due to not seeing the content of a container or a displacement of an object (Wimmer and Perner, 1983).

These *VS*-ER tasks, where children are also explicitly asked questions about someone’s beliefs, are generally passed by children at around the age of four (Wellman, 2014). However, recent research with infants challenges the assumption that this age represents a conceptual change in children’s social understanding. Non-verbal spontaneous-response (NV-SR) false belief tasks, which involve measuring infants’ looking times and anticipatory looking, suggest that infants and toddlers between 6 and 24 months can attribute false beliefs to other agents (Onishi and Baillargeon, 2005; Southgate et al., 2007; Surian et al., 2007; Kovács et al., 2010).

The origins and nature of the competence displayed by infants and children in NV-SR tasks have become the subject of a lively debate between viewpoints that deny or credit infants with a metarepresentational capacity. The associationist and constructivist perspectives, as explored by Heyes (2014) and Ruffman (2023), posit that infants’ reactions in false-belief tasks could be driven by basic associative processes or behavioral rules. Conversely, the mentalistic perspective, linked to researchers such as Leslie (1987), Carey and Spelke (1996), Baron-Cohen (1998), Carruthers (2011), and Scott et al. (2022), generally implies that individuals instinctively and non-verbally comprehend and interpret others’ mental states. Studying deaf children provides valuable evidence which contributes to this discussion, offering unique perspectives on the development of theory of mind in the absence of auditory input.

Previous research has reported that deaf children, particularly those who depend on spoken language, may encounter delays in comprehending false beliefs compared to their hearing counterparts. For instance, a study that compared deaf and hearing toddlers revealed that the deaf toddlers performed less effectively in a non-verbal false-belief task (Meristo et al., 2012). This research is significant as it underscores the potential influence of language and conversational interactions on the formation of theory of mind. It can enhance our understanding of the factors that impact the emergence and development of theory of mind in children and may have implications for interventions aimed at supporting false belief understanding in deaf children.

The purpose of the current study is to examine the performance of children with cochlear implants (CIs) on verbal and non-verbal false-belief tasks. By investigating the cognitive processes underlying their understanding of false beliefs, we aimed to explore whether the observed differences in performance between different tasks reflect reliance on language-based strategies.

### Verbal-story elicited-response false-belief tasks

Verbal-story elicited-response (*VS*-ER) tasks are widely used to assess false belief understanding (Wellman et al., 2001). In these tasks, a situation is presented where two agents possess different perspectives. For example, in the well-known change-of-location task, children are introduced to a story about two girls, Sally and Anne (Baron-Cohen et al., 1985). Sally places a marble in a basket and then leaves the scene. In her absence, Anne moves the marble from the basket to a box. The child is then asked where Sally will search for the marble upon her return. Passing the task requires understanding that Sally will search for the marble in the empty basket, demonstrating an understanding of her false belief. *VS*-ER tasks not only assess the child’s ability to attribute false beliefs but also tap into other developmental areas, such as executive functions and verbal ability.

Language development, including pragmatic, lexical, and grammar skills, has been found to be positively related to performance on these tasks (Milligan et al., 2007). Additionally, a child’s conversational environment, particularly the use of mental state language by mothers in conversations with their child, has been linked to the child’s performance on *VS*-ER tasks (Meins et al., 2002; Ruffman, 2023). This suggests that children’s performance on *VS*-ER tasks may be influenced by both their own language understanding and production as well as the conversational environment in which they grow up.

### Non-verbal spontaneous-response false-belief tasks

False-belief tasks administered to pre-verbal infants employ non-verbal methodologies and assess children’s spontaneous looking patterns as dependent measures (Onishi and Baillargeon, 2005; Song and Baillargeon, 2008; Senju et al., 2011; Surian and Geraci, 2012). These tasks involve presenting a story non-verbally, either through a computer screen or live, without requiring the child to comprehend or employ verbal language to pass the test. For example, in a study by Onishi and Baillargeon (2005), 15-month-old infants were shown an agent reaching for a watermelon in one of two boxes. Subsequently, the watermelon was moved to the second box while the agent either did or did not observe the relocation. The infants’ looking patterns indicated an expectation that the agent would reach for the watermelon in the location consistent with the agent’s false belief, rather than its actual location. These findings, along with numerous other studies, suggest an early capacity in infants to attribute beliefs to agents (Scott and Baillargeon, 2009).

### Deaf children with different conversational environments

Deaf children born into hearing families experience delays in the development of belief attribution skills when assessed using conventional verbal tasks (Peterson and Siegal, 1995; Meristo et al., 2007). The majority of deaf children are born to parents who do not use sign language and, as a result, these children lack access to language from birth. Growing up in a hearing environment limits their exposure to language-based communication and discussions about mental states, in contrast to their hearing peers. This limited conversational environment is believed to be the primary factor contributing to the delayed development of social perspective-taking skills (Woolfe et al., 2002). Research has shown that deaf children from deaf families, who are exposed to sign language from birth, follow a typical developmental trajectory, emphasizing the significance of environmental factors in social understanding (Woolfe et al., 2002; Meristo and Strid, 2020).

Currently, most deaf children receive cochlear implants (CIs) at an early age. However, studies examining the development of social perspective-taking abilities among children with CIs have produced mixed findings. Some studies indicate that these children demonstrate a disadvantage in *VS*-ER tasks compared to typically developing hearing children (Peterson, 2004; Walker et al., 2017; Akkaya and Doğan, 2023), while others suggest comparable performance between the two groups (Remmel and Peters, 2009) (also see Lundy, 2002; Macaulay and Ford, 2006; Moeller and Schick, 2006, for studies involving diverse samples of deaf children with and without CIs). In a recent study conducted by Meristo et al. (2016), preschool children with CIs were evaluated using both *VS*-ER and NV-SR tasks. The results indicated that they exhibited lower proficiency in the NV-SR task compared to hearing children, while performing equally well on the *VS*-ER. Conversely, deaf children from deaf families achieved scores comparable to those of their hearing peers (Meristo and Strid, 2020).

### The differences in performance on *VS*-ER and NV-SR tasks

Successful performance on *VS*-ER tasks requires the utilization of both verbal and non-verbal cognitive abilities, including advanced pragmatic and executive functioning skills. However, these abilities are not fully developed in infants before 4 years of age, which could explain their difficulty with such tasks. Interestingly, individuals with cochlear implants (Meristo et al., 2016) and those with Asperger syndrome (Senju et al., 2009) appear to find *VS*-ER tasks easier to solve compared to NV-SR tasks. One possible explanation for this difference is that traditional *VS*-ER tasks provide additional cues that facilitate task-solving (Bowler, 1992; Happé, 1995; Senju et al., 2009; Schneider et al., 2013). These cues might involve framing the verbal story in a mentalistic manner, explicitly directing attention to the agents’ mental states, or making the connection between mental states and actions more salient.

It has been suggested that high verbal ability could compensate for a lack of intuitive understanding of false belief problems in individuals with autism (Happé, 1995). For example, individuals with Asperger syndrome correctly answered false belief questions in Bowler’s (1992) study, but when asked to explain their solutions, they did so using logical terms, suggesting that traditional tasks can be solved at a non-mental level. More recent research indicates that adults with Asperger syndrome can often provide correct answers to traditional *VS*-ER tasks as well (Senju et al., 2009), despite experiencing difficulties in everyday social interactions. Thus, while typically developing infants may struggle with *VS*-ER tasks due to high verbal demands, linguistically advanced adults with Asperger syndrome may instead rely on embedded language cues to solve these tasks.

Another important distinction between *VS*-ER and NV-SR tasks is the amount of time available for deliberate reflection on others’ mental states. In *VS*-ER tasks, after the verbal question is presented (e.g., “Where will Sally look for her marble?”), children are not limited by short processing times to arrive at the correct answer. In contrast, NV-SR tasks do not involve traditional testing with verbal responses but require immediate and spontaneous responses. Bowler (1992) suggests that individuals with Asperger syndrome use a slower and more effortful approach to comprehend social situations, resulting in awkward everyday social interactions. Therefore, individuals with Asperger syndrome employ compensatory verbal and non-verbal cognitive abilities when performing on *VS*-ER tasks. Individuals lacking intuitive or spontaneous access to mental state understanding may rely on different strategies to correctly answer test questions in traditional verbal test settings. In other words, some participants’ success on traditional tasks may be attributed to problem-solving strategies that rely on verbal or attentional cues, rather than an intuitive and spontaneous ability to understand and reason about others’ mental states.

### The current study

The differences in performance between verbal and non-verbal tasks raise intriguing questions about the specific cognitive processes underlying the understanding of false beliefs in children with CIs. Does their struggle with non-verbal spontaneous-response (NV-SR) tasks imply a dependency on verbal narratives, or does it point to difficulties in spontaneously focusing on important details related to the mental states of individuals? To examine these possibilities, we introduced a novel non-verbal story elicited-response (NVS-ER) task (Pyers and Senghas, 2009). This task eliminates the need for participants to engage with any verbal narratives about the story character or respond to verbal questions. Instead, participants are presented with a series of cards arranged by the experimenter. The only language cue is when the experimenter verbally indicates that a card is missing and poses the question “Which one comes next?.” In this task, minimal language cues are provided, such as indicating a missing card and asking a simple verbal question. However, these cues are intentionally minimal and do not explicitly convey information about the character’s beliefs. They are designed to guide attention and prompt predictions based on the story sequence, rather than relying on language-based strategies. While present, these cues are insufficient to support an alternative strategy solely based on language. If children’s difficulties with the NV-SR task are due to the absence of an explicit question and the resulting need to rely on spontaneous mindreading, they should perform well on this new NVS-ER task. However, if the challenges with the NV-SR task primarily stem from its non-verbal nature, children should perform poorly also on the NVS-ER task.

## Method

### Participants

Twelve Estonian children with CIs participated (8 female, 4 male; mean age = 76 months, range 47–101 months; see Table 1) in our study. Eight children used bilateral and four children unilateral implants. The mean age of first implantation was 20 months (range: 16–33 months) and the children had used implants for the last 56 months on average (range: 28–76). Ninety-five per cent of parents had completed at least high school, and nine children had at least one sibling. None of the children with CIs had any deaf relatives, or native signers, in their immediate family. All children used spoken Estonian as their main way of communication at home. Eight children attended a bilingual school where classes were given either in spoken Estonian or ESL. These children had the freedom to select their preferred mode of communication, allowing them to switch between various subjects and school years. One child attended a deaf school where the mode of education was spoken Estonian, and three children were integrated in mainstream schools. All the children did not experience any language delays or deficiencies, except for the time prior to the implantation and adjustment of CI-s. The children did not have any known language deficiencies.

**Table 1:** Participants’ characteristics of the CI-group.

**Participant**	**Sex**	**CA**	**Age of CI**	**Non-verbal IQ**	**Vocabulary score**
Child 1	F	47	19	18	–
Child 2	F	59	17	12	68
Child 3	F	64	20	16	42
Child 4	M	67	16	21	78
Child 5	F	69	18	20	82
Child 6	M	72	19	29	125
Child 7	F	74	19	30	132
Child 8	F	85	18	23	131
Child 9	F	86	22	33	134
Child 10	M	96	21	21	96
Child 11	F	96	20	30	99
Child 12	F	101	33	28	84

The comparison group included 18 typically developing hearing Swedish children (nine female, nine male; mean age = 67 months, range 49–107). Ninety-four per cent of parents had completed high school and 16 children had at least one sibling. We did not add an additional comparison group of hearing children from Estonia as the societal contexts for children are very similar to Sweden.

We selected this specific age range because it aligns with the typical developmental stage when children begin to exhibit positive results in the *VS*-ER false belief tasks. Initially, we had reached out to parents of children with CIs in Sweden, but only two families agreed to take part in the study. Subsequently, we extended our outreach to the two deaf schools in Estonia and the Estonian organization for parents of children with cochlear implants. The current study comprises the families who agreed to participate.

All children were healthy and without known additional disabilities. The parents were informed about the purpose and procedure of the study and gave written consent. The data reported here were part of a larger study, the results of the *VS*-ER and NV-SR task have been previously reported in Meristo et al. (2016). The Regional Swedish Government Ethical Review Board and Tallinn Medical Research Ethics Committee in Estonia approved the study (Decision numbers: 484 and 1941).

### Measures

#### NVS-ER task

**Figure 1:**
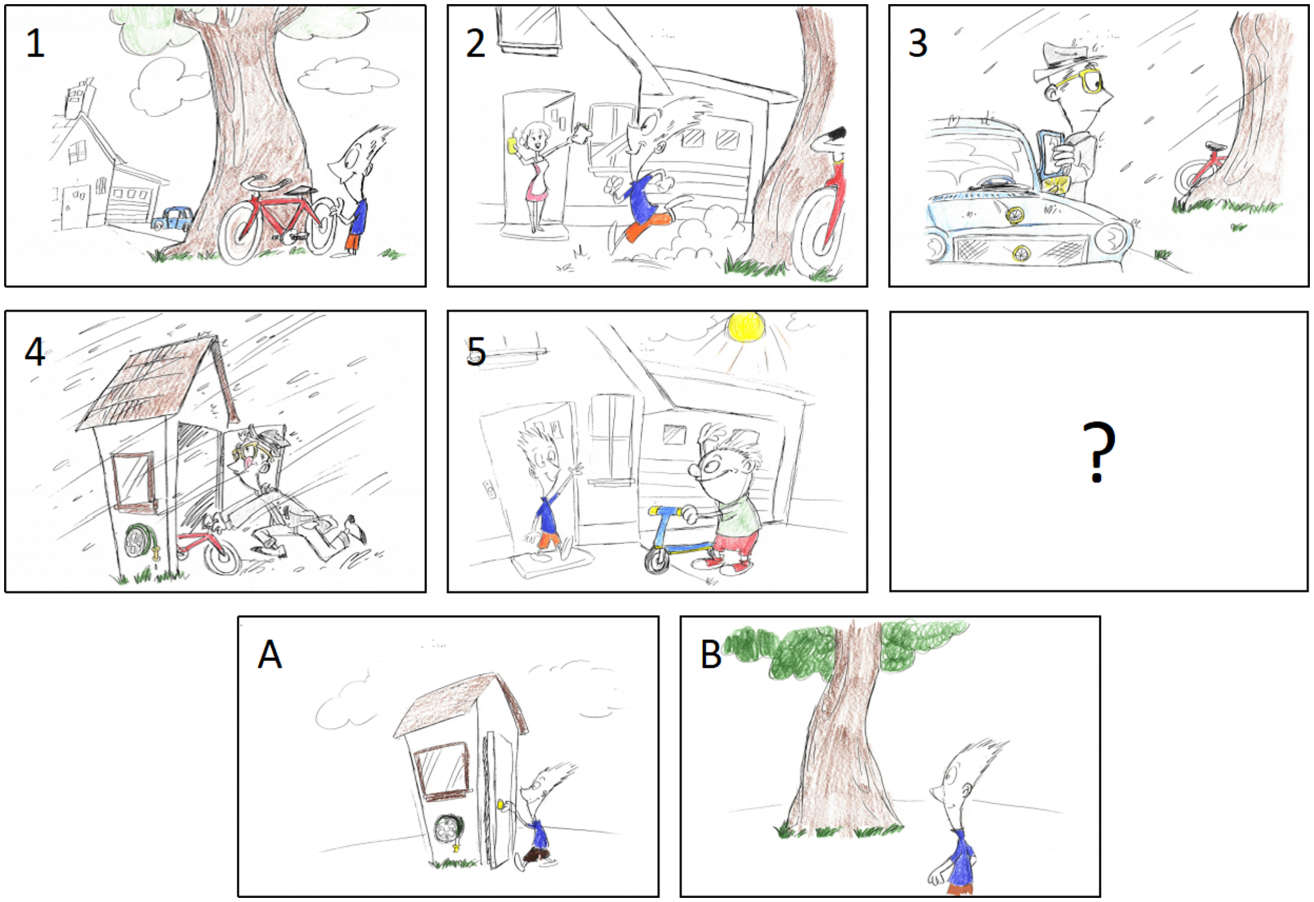
Examples of the story-card sequences (1–5) of the NVS-ER task.

An experimenter placed five cards (see Figure 1), one at a time, on the table in front of the child, making sure that the child looked at each picture before the experimenter proceeded to the next one. The pictures depicted a sequence of events. When all five cards were laid out, the experimenter pointed at each card in the correct order, making sure the child was attending to the cards. The experimenter then said “This card is missing” and pointed at the empty space next to the last card and put two new cards in front of the child asking “Which one comes next?” All children were presented with two training trials, to confirm that the child understood the procedure. If the child did not pick the correct card in the first training trial, the experimenter would explain which card was the correct one, and why. Both training trials were non-mentalistic stories and all children passed them. The three test trials, each consisting of five plus two cards, told stories in which one person left an object in one location and, when he or she was out of view, another person moved the object to another location. The two cards the child could choose from to finish the story showed the first person (1) searching for the object in its original location, or (2) searching for the object where it actually was. One score was given for the correct answer to each story (i.e., choosing the picture where the person was acting according to a false belief), leading to a maximum score of three. Children’s responses on both elicited-response tasks were documented by the experimenter and video recorded for *post hoc* coding by a second experimenter.

#### NV-SR task

**Figure 2:**
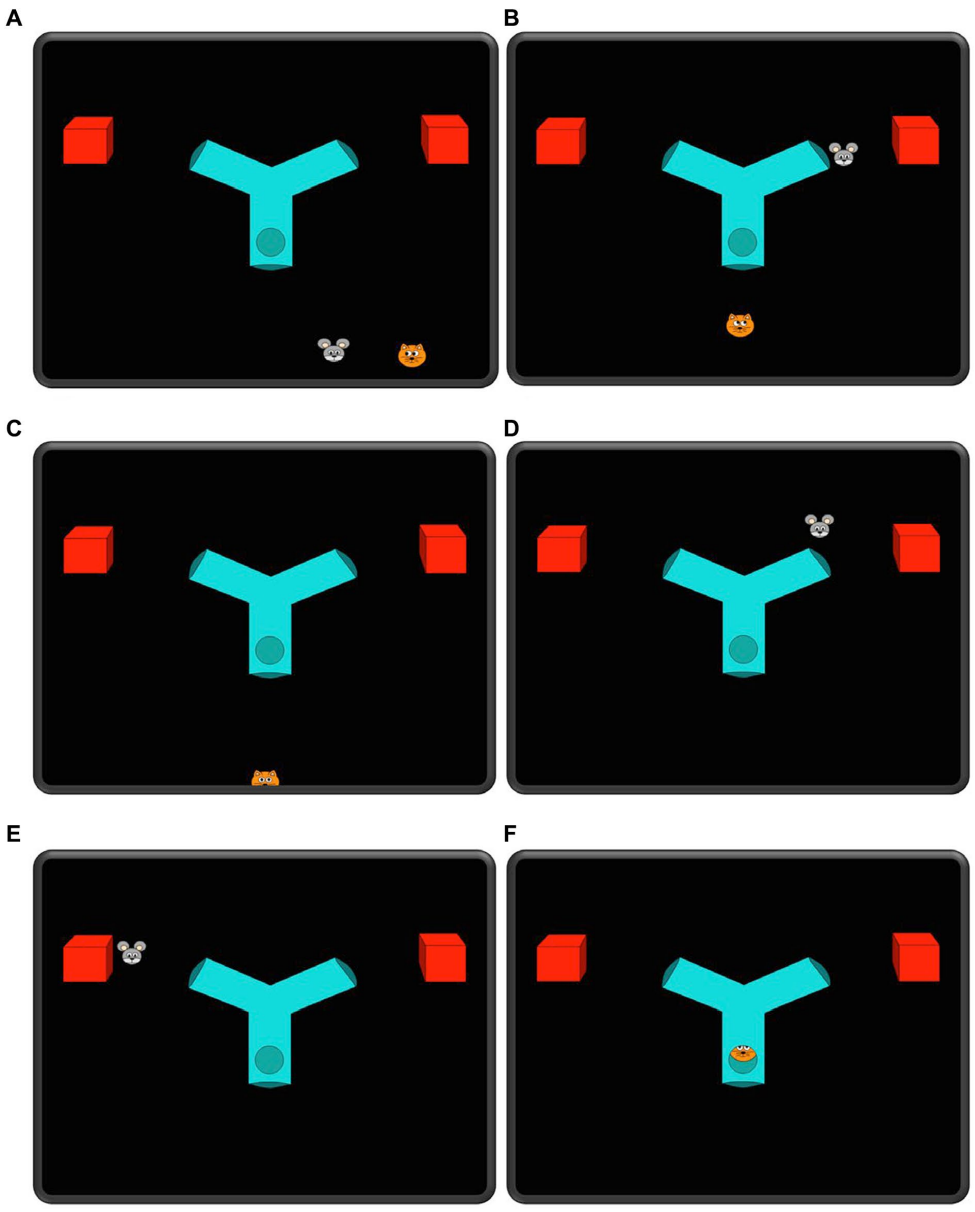
Selected frames (A–F) from the test event of the NV-SR task.

All children saw an animated movie (see Figure 2), adapted from Meristo et al. (2012) and Surian and Geraci (2012), and their eye gaze was measured with a Tobii near infrared eye tracker (T120). The child sat on their parent’s lap approximately 50–70 cm away from a 17-inch monitor. After a standard 5-point calibration, the child was presented with two familiarization trials and one test trial. In the familiarizations, the child saw a cat (Tom) follow a mouse (Jerry) into a Y-shaped tube with two exit points. There was one box outside each exit point. In the first familiarization, Jerry hid in one of the boxes and was followed by Tom and in the second familiarization, Jerry hid in the other box and was again followed by Tom (order of left and right exit was counterbalanced). The purpose of the familiarization was to establish that Tom would follow Jerry wherever he was hiding. Seven children (3 TH and 4 CI) were tested in addition but excluded from the final sample of this task since they did not anticipate the correct side for Tom’s appearance in the second familiarization. In the test trial Tom first watches when Jerry hides in one of the boxes and then Tom leaves the scene. While Tom is away, Jerry changes his hiding place to the other box and then Tom reenters and starts moving through the tube. The question of interest in the test trial was whether the child would expect Tom to search for Jerry in the box where he saw Jerry hide (i.e., behaving according to his false belief) or if Tom would search for Jerry where he actually was at that point. The child’s expectation was measured by calculating a differential looking score (DLS) (Senju et al., 2009) by subtracting looking to the incorrect exit (i.e., where Jerry was hiding) from looking to the correct exit (i.e., where Tom falsely believed the mouse was hiding), and by dividing it by the sum of time spent looking to any of the exits.

Children’s eye movements were recorded with a Tobii T120 (Tobii Technology, Sweden) near infrared eye tracker, and analyzed in Tobii Studio. Each child was seated in front of a 17-in. monitor placed 50–70 cm away. The children were told that “now we are going to look at a short movie” and instructed not to move during the task [either in Estonian Sign Language (ESL) or spoken language]. No other verbal instructions were given. Before the familiarization and the test trial, the children were given a 5-point calibration procedure represented by animated bouncing toys.

#### *VS*-ER task

Three tests were administered to each child. In the first one, the child was presented with two dolls (a boy and a girl), a ball, a basket and a box. The boy put the ball in the basket and then left the scene. While the boy was gone, the girl took the ball and put it in the box instead. The boy then returned and the child was asked “Where will the boy look for the ball?.” To pass the test, the child needed to answer the test question as well as two control questions (“Where is the ball really?” and “Where was the ball first of all?”) correctly. Two additional tests, with a different set of toys followed the identical procedure. Children who answered the test question and the control questions correctly earned one point for each test trial, with a total sum of zero to three points.

Additionally, children’s verbal ability was measured with the Peabody Picture Vocabulary Test (PPVT, Fourth Edition), the TH-group in spoken Swedish, and the CI-group in spoken Estonian (except one child who preferred ESL). Non-verbal IQ was measured with Raven’s Colored Progressive Matrices (RCPM). The two groups did not significantly differ on chronological age [*t*(28) = 1.36, *p* = 0.185, *d* = 0.51], non-verbal IQ [*t*(28) = 1.35, *p* = 0.189, *d* = 0.51] or Peabody picture vocabulary task [*t*(27) = 1.87, *p* = 0.072, *d* = 0.70] (see Table 2).

**Table 2:** Mean chronological age (in months), non-verbal IQ (Raven’s colored progressive matrices), Peabody Picture Vocabulary Test, and the results from the three kinds of false-belief tasks for each group.

**Group**	**TH-group**				**CI-group**				***p***
	**Mean**	**SD**	**Range**		**Mean**	**SD**	**Range**		
Chronological age (months)	67	18	49–107		76	17	47–101		0.185
Non-verbal IQ	19.8	7.5	9–35		23.4	6.5	12–33		0.189
Peabody vocabulary task	117.1	25.8	70–168		97.4	30.3	42–134		0.072
NVS-ER false belief task	1.94	0.80	1–3		1.75	0.97	0–3		
NV-SR false belief task	0.72	0.40	−0.21 - 1.00		0.16	0.65	−1.00 - 1.00		0.017*
*VS*-ER false belief task	2.5	0.8	1–3		1.8	1.4	0–3		

*p < 0.05.

### Procedure

All children were tested individually in a quiet room, at a school in Tallinn, Estonia, or at a research lab in Gothenburg, Sweden. The CI-children were tested in spoken Estonian by a hearing research assistant, except one child who preferred ESL and was tested by a deaf teacher (that is, she only consented to participate in the study when she learned that the deaf teacher, whom she was familiar with, would be the one conducting the tests. As a student at the bilingual school, she was proficient in ESL as well). The hearing children were tested in spoken Swedish by a research assistant. The teacher and research assistants were carefully instructed on how to administer the tasks. All test sessions were video recorded.

## Results

### Non-verbal-story elicited-response task

One point was credited for each correct selection of a picture that matched a false belief scenario in each story, with a maximum possible score of three. The dependent variable was the response accuracy, with a correct response coded as the baseline outcome. The binomial test was used to compare the number of correct responses out of three trials to the expected probability of guessing correctly by chance, which was 0.125 (12.5%) given two possible outcomes for each trial. In the TH group, 5 out of 18 children correctly responded to the test questions on all three trials (*p* = 0.032, one-tailed), whereas in the CI group 3 out of 12 children correctly responded to the test questions (*p* = 0.096, one-tailed).

We also applied a generalized linear mixed model to examine the effects of group membership and test occasions on the dichotomous outcome variable. The model included random intercepts for subjects to account for within-subject correlation across the three tests. The fixed effects revealed that the main effect of group membership was not statistically significant (*β* = −0.16, SE = 0.89, *p* = 0.86), nor were the main effects of the test occasions (Test 2: *β* = −0.82, SE = 0.76, *p* = 0.28; Test 3: *β* = −1.05, SE = 0.75, *p* = 0.16) or their interactions with group membership (Group × Test 2: *β* = −0.65, SE = 1.18, *p* = 0.58; Group × Test 3: *β* = 0.27, SE = 1.17, *p* = 0.82). The only significant finding was the intercept (*β* = 1.28, SE = 0.59, *p* = 0.03), suggesting a baseline effect. These results suggest that neither group membership nor test occasion significantly predicted the outcome when controlling for subject-level variability.

### Non-verbal spontaneous-response task

There was a significant difference in DLS between the groups [*t*(21) = 2.58, *p* = 0.017, *d* = 1.03], with the typical hearing children outperforming the children with CIs. The hearing children (*M* = 0.72, *SD* = 0.40), *t*(14) = 6.87, *p* < 0.001, *η*^2^ *=* 0.77, but not the children with CIs (*M* = 0.16, *SD* = 0.65), *t*(7) = 0.68, *p* = 0.519, *η*^2^ = 0.06, scored significantly above zero, that is they looked longer at the correct location. Three children in each group did not make a first anticipatory look toward one of the two relevant locations, that is the first fixation toward either the right or left exit of the tunnel from the moment the cat reemerges in search of the mouse. Thirteen of the 15 hearing children who made a first anticipatory look anticipated the reappearance at the correct FB location (*p* = 0.007, binomial test), compared to only 3 of 8 in the CI-group (*p* = 0.727).

### Verbal-story elicited-response task

In line with the NVS-ER task, a score was assigned for accurately responding to the false belief question in each story, with a maximum score of three. The response accuracy was the dependent variable, and a binomial test was employed to evaluate the number of correct responses from three trials against the probability of correctly guessing by chance, which was set at 0.125 (or 12.5%), considering there were two possible outcomes for each trial. The results showed that both the TH-children (12 out of 18 children, *p* < 0.001, one-tailed) and the CI-children (6 out of 10 children, *p* < 0.001, one-tailed) correctly responded to the test questions on all three trials.

We also employed a GLMM repeated measures design to investigate the influence of group (TH-children vs. CI-children) and the three different test trials, each a dichotomous variable. The main effect of group membership was not significant (*β* = 7.15, SE = 4.20, *p* = 0.09). The main effect for the third test occasion was significant (*β* = 5.57, SE = 2.61, *p* = 0.03), but not the second test occasion (*β* = 2.43, SE = 1.91, *p* = 0.20). Notably, the interaction effects between group membership and the second (*β* = −9.88, SE = 4.45, *p* < 0.03) and third (*β* = −13.02, SE = 4.94, *p* < 0.01) test occasions were both significant, suggesting that the effect of group membership on the outcome varied across the tests. These findings suggest that the likelihood of passing the false-belief tests varied between the typically hearing children and those with cochlear implants, and this effect was not consistent across the tests.

## Discussion

Previous studies have indicated that toddlers, preschoolers, and school-age children with cochlear implants (CIs) face challenges in spontaneously attributing false beliefs to others, a skill usually observed in typically developing preverbal infants (Meristo et al., 2016). However, children with CIs appear to follow a typical developmental trajectory in traditional verbal false belief tasks like the Sally and Anne task (Meristo et al., 2016; Pluta et al., 2021). Despite their exposure to social language-based interactions with family and friends after CI implantation, they still struggle with NV-SR tasks. To address this question, we conducted a comparative study involving children with CIs and a hearing comparison group, employing three different types of false belief tasks.

Our results revealed that children with CIs demonstrate strong performance in verbal-story elicited-response (*VS*-ER) tasks, indicating their ability to anticipate others’ actions based on beliefs when supported by verbal narratives. However, their performance does not surpass chance levels when asked to form belief-based inferences without explicit verbal cues, as in non-verbal-story elicited-response (NVS-ER) or non-verbal-story spontaneous-response (NV-SR) tasks. Tasks that require spontaneous responses limit children’s time for deliberate reflection on others’ mental states. Eliminating this time constraint in the non-verbal-story elicited-response (NVS-ER) task did not lead to successful performance for children with CIs, indicating that cognitive challenges associated with these tasks cannot be overcome simply by easing the cognitive load. In the non-verbal-story elicited-response (NVS-ER) task, children were presented with a complete storyline through pictures. They had the flexibility to go back and forth among the different images at their own pace, giving them ample time to select the appropriate response. Additionally, this task did not entirely rule out the potential use of logical problem-solving or internalized language-based strategies. Thus, our findings indicate that children with CIs find it challenging to solve false-belief tasks without external narrative support. This highlights the importance of external cues and linguistic narratives in facilitating social cognition in children with CIs. By providing explicit verbal information, narratives can help children frame the verbal story in a mentalistic manner, explicitly directing attention to the agents’ mental states, or making the connection between mental states and actions more salient.

It is important to recognize that the varying performance on the three false-belief tasks may have been influenced by factors beyond their reliance on language. The significant task differences, such as the animacy of the moved target (bike vs. Jerry vs. apple), the use of human vs. cartoon vs. puppet as the main character, and the presentation of picture cards vs. abstract cartoons vs. toys, have the potential to impact perspective taking. It is crucial to consider these task differences when interpreting the findings. However, previous research on the NV-SR tasks has employed diverse setups, including the use of cartoon characters (Surian and Geraci, 2012), human characters (Onishi and Baillargeon, 2005; Southgate et al., 2007), and live events with humans (Barrett et al., 2013). Additionally, Setoh et al. (2016) developed a NVS-ER task using pictures with human characters and obtained favorable results with 2.5-year-olds. Similarly, decades of research on the *VS*-ER task (Wellman et al., 2001) have utilized a variety of setups. These findings suggest that both verbal and non-verbal tasks demonstrate robustness and independence from specific contextual factors.

In typical anticipatory looking tasks, participants are initially presented with two familiarization trials involving goal-directed actions to familiarize them with the setup before a false belief-based test trial is introduced. The participants’ ability to anticipate a goal-directed action in these trials serves as a crucial validity check for this paradigm. However, seven children, constituting 23% of the total group (33% of the CI-group, and 17% of the TH group), failed to anticipate these goal-directed actions in our familiarization trials. Consequently, they were excluded from the NV-SR task analysis. This exclusion rate aligns with what is usually observed in anticipatory looking false-belief tasks involving typically developing hearing children (Steffan et al., 2023). As a result, it is unlikely that the lower performance of CI-children on the NV-SR task can be attributed to challenges in understanding the task’s setup or comprehending others’ goals and intentions.

In recent years, concerns have been raised about the replicability of anticipatory looking measures in the field (Kaltefleiter et al., 2022). These concerns include a self-non-replication of a pivotal study (Southgate et al., 2007) that laid the groundwork for notable findings involving individuals with Asperger syndrome (Senju et al., 2009; Kampis et al., 2021). The specific stimuli used in our method, adapted from Surian and Geraci (2012), have also faced debates concerning their replicability (Kulke et al., 2018). Given these replication issues, caution should be exercised when interpreting results from studies utilizing anticipatory looking measures. While our study primarily focused on task design and language cues, it is crucial to address the potential impact of replication issues on result interpretation. We acknowledge the need for further research to investigate the replicability of these measures and their potential influence on outcomes.

### Limitations

This study is subject to several limitations that should be taken into account when interpreting the results. First, the study’s limited sample size poses a significant constraint on the extent to which the findings can be generalized to the broader population of children with cochlear implants (CIs). With a larger sample size, the study would have greater statistical power, allowing for a more comprehensive analysis of the data and enhancing the reliability of the findings. Second, due to the small sample size, the statistical robustness of the findings is limited. While the results are suggestive, they should be interpreted with caution and confirmed by future research with larger sample sizes. For example, the performance of the TH group on the NVS-ER task was not too different from the CI group given that only 5 out of 18 children in the TH group scored 3 out of 3 and this result was significant only at a one-tailed 0.03 significance level. Third, it is important to emphasize that this paper is exploratory in nature, given the small sample size for children with CIs. The findings should be considered preliminary and in need of replication with larger samples to establish their generalizability to the broader population of children with CIs. Finally, looking time measures can offer valuable insights into cognitive processes, but their interpretation is a subject of controversy, as demonstrated by the different theoretical perspectives presented in the introduction (Heyes, 2014; Wellman, 2014; Tomasello, 2018; Ruffman, 2023). For example, some authors argue that infants’ false belief tasks in looking time studies can be interpreted as their understanding of epistemic states such as knowledge/ignorance rather than false beliefs. There is however considerable evidence about infants’ understanding of true and false beliefs in their second year of life (for review see Baillargeon et al., 2010).

## Conclusion

In the current study, we found that children with CIs experience difficulties in performing well in non-verbal false belief tasks, both spontaneous-response and elicited-response tasks, while performing well in verbal tasks. Our results underscore the significance of external narrative support for children with CIs in tasks requiring action prediction based on false beliefs. Children with CIs often face challenges in fully participating in conversations, which affects their ability to attend to relevant information and infer mental states accurately. However, it is important to interpret these conclusions with caution, as further research is needed to fully understand the complex nature of belief attribution in children with CIs. Additional studies that investigate a range of factors, including language proficiency, cognitive abilities, and social experiences, can provide a more comprehensive understanding of the underlying mechanisms involved.

## Data availability statement

The raw data supporting the conclusions of this article will be made available by the authors, without undue reservation.

## Ethics statement

The Regional Swedish Government Ethical Review Board and Tallinn Medical Research Ethics Committee in Estonia approved the study (Project title: Development of social cognition in hard-of-hearing children; Decision numbers: 484 and 1941). The studies were conducted in accordance with the local legislation and institutional requirements. Written informed consent for participation in this study was provided by the participants’ legal guardians/next of kin.

## Author contributions

MM designed the study, and prepared the experimental materials. MM and KS carried out the data collection and the statistical analyses, and wrote the first draft of the manuscript. MM and LS provided the revisions. All authors contributed to the article and approved the submitted version.
